# Mid-term outcomes of modified valve-sparing aortic root replacement versus the Bentall procedure for middle-aged Chinese patients with acute DeBakey I aortic dissection: a single-center retrospective study

**DOI:** 10.1186/s12872-021-02014-5

**Published:** 2021-04-20

**Authors:** Qingsong Wu, Zhisheng Wang, Zhihuang Qiu, Yue Shen, Xiaodong Chen, Xingfeng Chen, Liangwan Chen

**Affiliations:** 1 Department of Cardiac Surgery, Union Hospital, Fujian Medical University, Fuzhou, 350001 Fujian People’s Republic of China; 2 Fujian Key Laboratory of Cardio-Thoracic Surgery (Fujian Medical University), Fuzhou, 350001 Fujian People’s Republic of China; 3 The Affiliated Longyan First Hospital of Fujian Medical University, Longyan, 364000 Fujian People’s Republic of China

**Keywords:** Acute DeBakey I aortic dissection, Middle-aged patients, Aortic root surgery, Endoleakage, Thromboembolism/bleeding events, Re-operation

## Abstract

**Background:**

The mid-term and long-term efficacies of valve preservation in acute DeBakey I aortic dissection (AD) are controversial. Thus, it is unclear whether middle-aged patients with acute DeBakey I AD should undergo modified valve-sparing procedures or the Bentall procedure in an emergency setting.

**Methods:**

This study included 213 middle-aged Chinese patients (under 60 years old) who were treated for acute DeBakey I AD between January 2009 and June 2015. The treatments involved modified valve-sparing aortic root replacement (VSARR) (117 patients) or the Bentall procedure (96 patients). Preoperative, intraoperative, postoperative, and follow-up data were analyzed. Echocardiography and thoracoabdominal computed tomography angiography (CTA) findings were reviewed at 3 months, 1 year, and then annually after surgery.

**Results:**

No significant differences were observed in terms of the preoperative, intraoperative, in-hospital mortality, and postoperative parameters. There were also no significant differences in aortic regurgitation (AR). However, follow-up examinations using CTA revealed that 6 patients had endoleakage at the distal end of the triple-branched stent (0/110 patients [0.0%] vs. 6/90 patients [6.7%], *P* = 0.022). The anticoagulation-related thromboembolism/bleeding events was significantly lower in group A than in group B (0/110 patients [0.0%] vs. 11/90 patients [11.1%], *P* = 0.001). And postoperative aortic valve re-operation rate was significantly lower in group A (1/110 patients [0.9%] vs. 8/90 patients [8.9%], *P* = 0.020). There was no significant difference in survival during the follow-up period (log-rank *P* > 0.05).

**Conclusion:**

For middle-aged patients with acute DeBakey I AD, VSARR were associated with a lower rate of endoleakage at the distal end of the triple-branched stent, thromboembolism/bleeding events and aortic valve re-operation (vs. the Bentall procedure).

## Introduction

The rapid development of China's social economy has improved the national standard of living and medical care. These changes have helped decreased the case fatality and disability rates, which have led to increasing life expectancy. Thus, China’s healthcare system is facing increasing challenges [1], especially in terms of managing and follow-up postoperative patients of AD. Aortic dissection (AD) is deadly and its treatment is challenging for cardiovascular surgeons. In particular, some provinces in China with underdeveloped medical conditions. And in China, the majority of AD patients are relatively young. The occurrence of AD typically involves distal extension of a medial tear in the aortic wall, as well as proximal extension to the aortic root, which leads to aortic sinus dilation, aortic valve insufficiency, and coronary artery dissection [2, 3]. Severe aortic insufficiency can subsequently lead to acute left heart failure, cardiac tamponade, increased surgical risk, and postoperative complications and mortality [4]. Several strategies can be used to preserve the aortic valve, although these strategies remain technically challenging and prone to complications [5, 6].

However, it is unclear whether middle-aged patients with acute DeBakey I AD should undergo valve-sparing procedures or the Bentall procedure in an emergency setting. Therefore, this study aimed to investigate retrospectively mid-term effects of the valve-sparing procedures and the Bentall procedure in an emergency setting in middle-aged patients with acute DeBakey I AD.

## Methods

### Study population

This single-center retrospective study evaluated 213 middle-aged Chinese patients (under 60 years old) who underwent treatment for acute DeBakey I AD between January 2009 to June 2015. The treatments involved VSARR with ascending aortic and hemiarch replacement and triple-branched stent implantation (Group A, 117 patients) or the Bentall procedure with hemiarch replacement and triple-branched stent implantation (Group B, 96 patients).The triple-branched stent graft used in this study were manufactured by Yuhengjia Sci Tech Corp Ltd, Beijing, China [7]. All patients underwent emergency surgery.

We included: (1) patients who were middle-aged under 60 years old, (2) patients with acute DeBakey I AD and underwene emergency surgery. And we excluded: (1) patients with a history of heart surgery, (2) patients with Marfan syndrome, aortitis, metoxoarteritis, or systemic immune diseases, (3) patients with preoperative infective endocarditis, malignant tumors, or chronic organ failure.

### Definitions

For the present study, patients were considered a middle aged patient is under 60 years old. Aortic insufficiency was defined as AD involving the sinus of Valsalva that led to dilation and insufficiency. VSARR involved a modified David procedure that incorporated the patch neointima technique [8]. The Bentall procedure was defined as the replacement of the aortic root with a composite valve-graft device (Bioprosthetic valve, Medtronic Inc. USA or Edwards Lifesciences LLC. USA. Mechanical valve, Sorin Group, Milan, Italy S.r.l or Beijing Sta Escending Equipment Co., Ltd. GKS CHN) [9]. Anticoagulation-related thromboembolism/bleeding events include cerebral hemorrhage, gastrointestinal hemorrhage, mechanical flap stuck event, cerebral infarction, myocardial infarction,because of coagulation function was abnormal.

### Study parameters

The study parameters included the patients’ baseline and preoperative characteristics, operative data, postoperative outcomes, and follow-up data. Baseline and preoperative characteristics included age, sex, body mass index, time between symptom and surgery, smoking history, hypertension, diabetes mellitus, coronary heart disease, coronary malperfusion, shock, cerebral malperfusion, extremity malperfusion, acute liver failure, acute renal failure, and acute left heart failure, moderate or severe AR, left ventricular end diastolic diameter and EF. Operative parameters included the operative time, cardiopulmonary bypass (CPB) time, aortic clamp time, selective brain perfusion (SBP) time, intraoperative blood loss, and intraoperative red blood cell (RBC) infusion. Postoperative outcomes include pericardial drainage volume during the 24 h after surgery, re-thoracotomy for hemostasis, mechanical ventilation, admission to the intensive care unit, hospitalization time, early death (< 30 days after surgery), and major adverse outcomes, such as transient/permanent neurological complications, gastrointestinal hemorrhage, acute renal failure, acute liver failure, sepsis, spinal cord injury and concomitant procedure. Follow-up data were collected regarding death, anticoagulation-related thromboembolic/bleeding events, mechanical disturbance of the valve, perivalvular leakage, valve aging, infection, endoleakage at the distal end of the triple-branched stent, and postoperative aortic valve re-operation.

### Surgical procedures

**Fig. 1:**
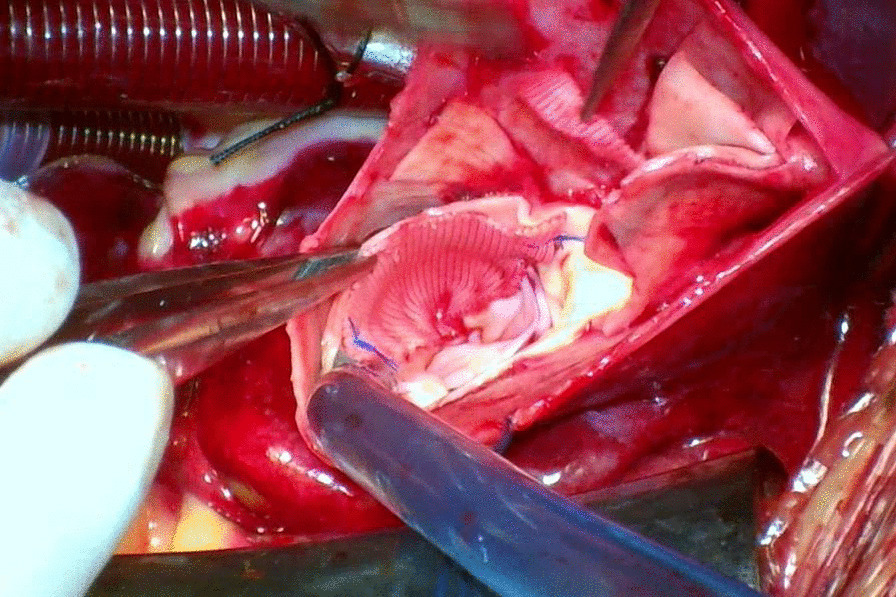
In the noncoronary sinus, a vertical 3–0 polypropylene continuous suture was placed internally from the base of the sinus toward the transected edge of the aortic root

**Fig. 2:**
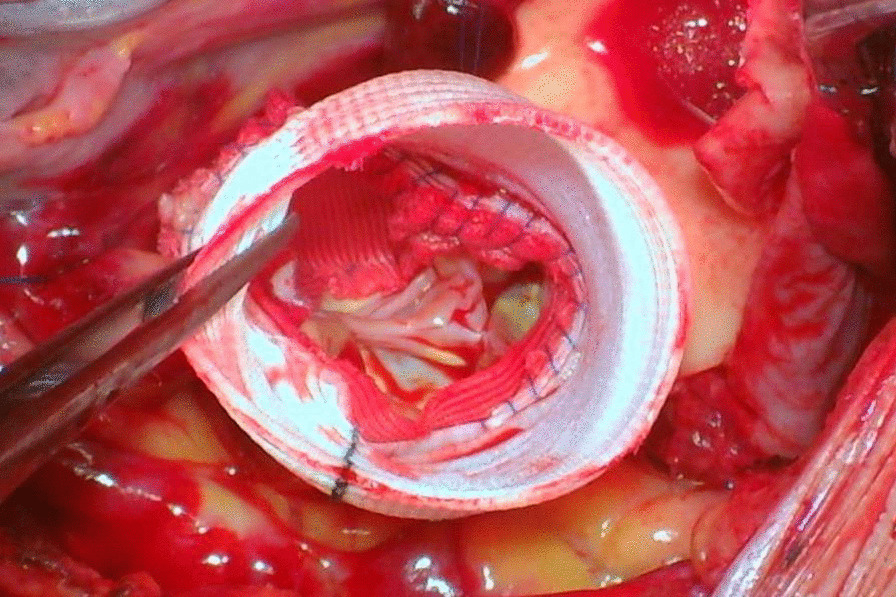
The modified valve-sparing aortic root technique using patch neointima technique

The detailed of VSARR has been described previously [8, 10]. All operations were performed under general anesthesia with CPB, and intraoperative esophageal echocardiography was routinely performed. After CPB was established, the ascending aorta was clamped at the brachiocephalic root, the ascending aorta were dissected. The VSARR was used to repair the aortic valve of patients with severe AR caused by the dilatation of the aortic sinus. We cut three tear-drop patches (polyester surgical patch; Chest Medical Science and Technology Ltd., Shanghai, China) to match the size and shape of the aortic sinus. (Fig. 1) A continuous suture was placed on the medial side of the aortic sinus and small holes were made in the left and right coronary sinus patches, which were then sutured and anastomosed along the surrounding sinus wall of the in-situ coronal openings. Reconstruct the ascending aorta via anastomosis with the aortic root to complete the VSARR (Fig. 2).

Circulatory arrest began when the rectal temperature dropped to 22–25 ℃. A right axillary cannula was used to establish antegrade SBP (10–15 mL/kg/min). The triple-branched stent was implanted to reconstruct the aortic arch. Finally, we anastomosed the distal end of the Dacron tube graft and the proximal end of the triple-branched stent graft, restored full-flow perfusion, and completed the operation [11, 12].

### Follow-up

Patients who survived to discharge underwent outpatient follow-up via telephone or message-based communication. All patients also underwent follow-up examinations using echocardiography and thoracoabdominal CTA at 3 months, 1 year, and then annually after surgery. All patients received oral warfarin anticoagulation after the Bentall surgery. Patients were followed until death because of any cause or loss to follow-up.

### Statistical analysis

Continuous variables were presented as mean ± SD or medium (Q25, Q75) and categorical variables were presented as numbers (%). Inter-group differences were evaluated using the t test or Mann–Whitney U for continuous variables, and using the chi-squared test for categorical variables. Survival analyses were performed using the Kaplan–Meier method and log-rank test was used to calculate the P value. All analyses were performed using IBM SPSS software (version 19.0; IBM Corp., Armonk, NY, USA) or R (version 3.6.0 Auckland University, New Zealand). All differences were considered statistically significant at two-tail *P* values of < 0.05.

## Results

The 213 eligible patients included 117 patients who underwent VSARR with ascending aortic and hemiarch replacement and triple-branched stent implantation (Group A) and 96 patients who underwent the Bentall procedure with hemiarch replacement and triple-branched stent implantation as the control group (Group B). No significant differences were observed in the two groups’ baseline and preoperative characteristics (Table 1).

**Table 1:** Preoperative data on the two patient groups

Valuables	Group A (n = 117)	Group B (n = 96)	*P* value
Age (years)	51 (47, 55)	51 (45, 55)	0.609
Male gender (%)	77 (65.8)	68 (70.8)	0.434
Body mass index (kg/m^2^)	24.9 (23.2, 27.7)	24.7 (23.0, 27.3)	0.402
Time from onset of symptoms to surgery (h)	26 (16, 34)	27 (18.3, 40.5)	0.201
Smoking history (%)	60 (51.3)	54 (56.3)	0.469
Hypertension (%)	97 (82.9)	80 (83.3)	0.934
Diabetes mellitus (%)	4 (3.4)	2 (2.1)	0.865
Coronary heart disease (%)	2 (1.7)	2 (2.1)	0.759
Preoperative complications			
Coronary malperfusion (%)	0 (0.0)	2 (2.1)	0.117
Shock (%)	1 (0.9)	2 (2.1)	0.449
Cerebral malperfusion (%)	0 (0.0)	1 (1.0)	0.268
Extremity malperfusion (%)	2 (1.7)	2 (2.1)	0.759
Acute renal failure (%)	4 (3.4)	3 (5.2)	0.790
Acute liver failure (%)	2 (1.7)	2 (2.1)	0.759
Acute left heart failure (%)	2 (1.7)	4 (4.2)	0.508
AR (moderate or above (%)	112 (95.7)	96 (100)	0.111
Aortic root true lumen diameter (mm)	40.0 (38.0, 42.0)	40.0 (36.9, 44.2)	0.585
Left ventricular diastolic diameter (mm)	46.2 (43.0, 52.6)	48.0 (44.7, 51.0)	0.771
EF (%)	65.0 (60.1, 67.6)	64.2 (60.7, 67.1)	0.614

Continuous variables were present as median (Q25, Q75). Wilcoxon rank test for continuous variables and chi-square test for categorical variables

### Operative data

Table 2 shows the operative data for the two groups. No significant differences were observed in terms of operative time, CPB time, AC time, SBP time, intraoperative blood loss, and intraoperative RBC infusion. All patients who underwent VSARR were intraoperatively evaluated using esophageal echocardiography, which revealed satisfactory repair results. 11 patients had trace aortic AR and 4 had mild AR.

**Table 2:** Surgical data on the two patient groups

Valuables	Group A (n = 117)	Group B (n = 96)	*P* value
Operative time (min)	282 (264, 350)	288 (255, 339)	0.686
CPB time (min)	136 (110, 166)	127 (112, 153)	0.418
aortic clamp time (min)	57.0 (47.5, 66.0)	55.0 (48.3, 76.8)	0.737
SBP time (min)	20.0 (18.0, 24.0)	20.5 (19.0, 23.0)	0.811
Intraoperative blood loss (mL)	300 (200, 400)	300 (200, 300)	0.434
Intraoperative red blood cell infusion (UL)	3.0 (2.0, 4.0)	3.0 (2.0, 4.0)	0.617
**Valve operation procedure**			
Bentall with mechanical valve (%)	0 (0.0)	93 (96.9)	N/A
Bentall with Bioprosthetic valves (%)	0 (0.0)	3 (3.1)	N/A
VSARR (%)	117 (100.0)	0 (0.0)	N/A
**Concomitant procedure**			
Coronary artery bypass grafting	2 (1.7)	2 (2.1)	0.759
Mitral valve replacement	1 (0.9)	2 (2.1)	0.449
Tricuspid valvuloplasty	0 (0.0)	1 (1.0)	0.268

Continuous variables were present as median (Q25, Q75) or mean ± SD. Chi-square test for categorical variables and t test or wilcoxon rank sum test for continuous variables

### Early mortality and morbidity

There was no significant difference in terms of early death between group A and group B (6/117 patients[5.1%] vs. 5/96 patients[5.2%], *P* > 0.05). We did not encounter any bleeding events involving the aortic anastomoses. Postoperative complications and morbidity data are summarized in Table 3. There were no significant inter-group differences in terms of pericardial drainage volume during the 24 h after surgery, re-thoracotomy for hemostasis, mechanical ventilation, admission to the intensive care unit, or hospitalization time. Comparison of groups A and B also revealed no significant differences in low cardiac output syndrome (1/117 patients [1.7%] vs. 0/96 patients [0.0%], *P* > 0.05), transient neurological complications (7/117 patients [6.0%] vs. 5/96 patients [3.1%], *P* > 0.05), permanent neurological complications (0/117 patients [0.0%] vs. 2/96 patients [1.0%], *P* > 0.05), gastrointestinal hemorrhage (1/117 patients [0.0%] vs. 4/96 patients [4.2%], *P* > 0.05), acute renal failure (4/117 patients [3.4%] vs. 4/96 patients [4.2%], *P* > 0.05), or sepsis (1/117 patients [0.8%] vs. 3/96 patients [3.1%], *P* > 0.05). No cases of acute liver failure or spinal cord injury were identified.

**Table 3:** Postoperative complication and morbidity data on the two patient groups

Valuables	Group A (n = 117)	Group B (n = 96)	*P* value
Early death (%)	6 (5.1)	5 (5.2)	0.776
Low cardiac output syndrome (%)	1 (0.8)	0 (0.0)	0.921
Re-thoratomy for hemaostsis (%)	1 (0.8)	2 (2.1)	0.863
Pericardial drainage volume in 24 h after surgery (mL)	500 (350, 600)	350 (250, 575)	0.668
Mechanical ventilation (h)	52.0 (44.0, 85.0)	55.0 (41.0, 83.0)	0.747
Intensive care in ICU (d)	4.0 (32.0, 73.0)	4.0 (29.0, 71.0)	0.256
Hospitalization time (d)	17 (15, 24)	18 (14, 21)	0.403
Gastrointestinal hemorrhage (%)	1 (0.8)	4 (4.2)	0.257
Acute kidney failure (%)	4 (3.4)	4 (4.2)	0.939
Acute liver failure (%)	0 (0.0)	0 (0.0)	N/A
Sepsis (%)	1 (0.8)	3 (3.1)	0.479
Transient neurological complications (%)	7 (6.0)	5 (5.2)	0.935
Permanent neurological complications (%)	0 (0.0)	2 (2.1)	0.393
Spinal cord injury (%)	0 (0.0)	0 (0.0)	N/A

Continuous variables were present as median (Q25, Q75) or mean ± SD. Chi-square test for categorical variables and t test or wilcoxon rank sum test for continuous variables

### Follow-up data

**Fig. 3:**
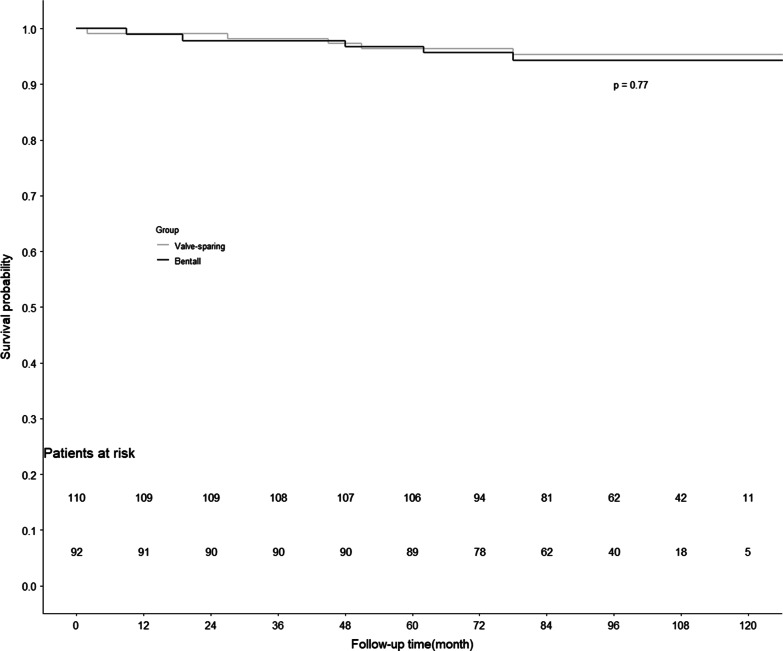
Survival curve 1

The mean follow-up durations were similar between discharged patients (n = 202) who underwent the valve-sparing and Bentall procedures (95.9 ± 22.7 months vs. 91.2 ± 20.8 months, *P* = 0.130), and survival data were available for all patients except 2 patient (1.0%) who was lost to follow-up. In group A, 1 year cumulative survival 99.1% and the cumulative 5-year survival rate was 96.4%. While in group B, 1 year cumulative survival 98.9% and the cumulative 5-year survival rate was 95.7%. The survival curves are shown in Fig. 3. 5 patients (4.5%) in group A died, including 1 patient died from multiple organ dysfunction with follow-up for 2 months. 1 patient died from traffic accident were followed for 27 months. Another causes of death in group A were shock (1 patients) with follow-up for 45 months, acute myocardial infarction (1 patients) and severe lung infection (1 patient) with follow-up for 51 months and 78 months. 5 people (5.6%) in group B died, and these deaths were related to low cardiac output syndrome and sepsis after re-operation, myocardial infarction, hemorrhage of brain stem and infective endocarditis (1 case each).

In group A, there were no anticoagulation-related thromboembolism or bleeding events after the operation. 8 patients developed mild aortic valve regurgitation, and 2 patients developed modarate regurgitation. All 10 patients had undergone echocardiography, which revealed trace AR at hospital discharge, and were in good condition during the follow-up. 2 patients in group B experienced cerebral hemorrhage (testing revealed a prothrombin time of up to 84 s) and one developed hemiplegia after craniotomy to remove the hematoma, and the other died from hemorrhage of brain stem. 3 patients developed mechanical valve disturbance, two of them led to acute left heart failure, and underwent emergency surgical treatment ultimately died because of acute postoperative low cardiac output syndrome. The other survived due to the used of extracorporeal membrane oxygenation before the second surgical. Follow-up examinations using CTA revealed that 6 patients had endoleakage at the distal end of the triple-branched stent graft (0/110 patients [0.0%] vs. 6/90 patients [6.7%], *P* = 0.022). The Anticoagulation-related thromboembolism/bleeding events was significantly lower in group A than in group B (0/110 patients [0.0%] vs. 11/90 patients [11.1%], *P* = 0.001). And the aortic valve re-operation rate was significantly lower in group A than in group B (1/110 patients [0.9%] vs. 8/90 patients [8.9%], *P* = 0.020) (Table 4).

**Table 4:** Follow-up data on the two patient groups

Valuables	Group A (n = 110)	Group B (n = 90)	*P* value
Follow-up time (months)	95.9 ± 22.7	91.2 ± 20.8	0.130
Cerebral hemorrhage (%)	0 (0.0)	3 (3.3)	0.191
Cerebral infarction (%)	0 (0.0)	1 (1.1)	0.948
Gastrointestinal hemorrhage (%)	1 (0.9)	4 (4.4)	0.268
AR (moderate or above) (%)	2 (1.8)	0 (0.0)	0.590
Perivalvular leakage (%)	0 (0.0)	1 (1.1)	0.948
Biological valve aging (%)	0 (0.0)	0 (0.0)	N/A
Infection (%)	1 (0.9)	2 (2.2)	0.879
Mechanical disturbance of the valve (%)	0 (0.0)	3 (3.3)	0.191
endoleakage (%)	0 (0.0)	6 (6.7)	**0.022**
Anticoagulation-related thromboembolism/bleeding events* (%)	0 (0.0)	10 (11.1)	**0.001**
Postoperative aortic valve re-operation (%)	1 (0.9)	8 (8.9)	**0.020**

Continuous variables were present as median (Q25, Q75) or mean ± SD. Chi-square test for categorical variables and t test or wilcoxon rank sum test for continuous variables

Bold letters indicate a statistically significant difference

*Anticoagulation-related thromboembolism/bleeding events include cerebral hemorrhage, gastrointestinal hemorrhage, mechanical flap stuck event, cerebral infarction, myocardial infarction, because of coagulation function was abnormal

## Discussion

The rapid development of acute DeBakey I AD is associated with a high risk of mortality and necessitates emergency surgery, which can significantly reduce the mortality rate [13]. There are two classic and commonly used methods for treating dissection that involves the aortic root, which are the Bentall procedure and the David procedure. Thus, the choice of treatment is based on whether the aortic root is involved, the course of the disease, and the surgeon's expertise [14–17].

The Bentall procedure is simpler and shorter than the David procedure, but all patients received oral warfarin anticoagulation after surgery. We encountered 3 patient who developed mechanical valve dysfunction, and two of them led to acute left heart failure, and one patient subsequently died because of low cardiac output syndrome after emergency surgery. In this setting, thrombus abscission from the mechanical valve can lead to cerebral infarction, myocardial infarction, and other embolic events. It can also led to gastrointestinal hemorrhage, cerebral hemorrhage and other warfarin—related bleeding events. Valve aging of biovalves is another concern, especially in patients with diabetes, uremia, and other complications, which can accelerate the valve aging processand lead to sclerosis, calcification, and contracture at the biovalves [18]. Moreover, the proliferation of tissue around the valvecan lead to the narrowing of the left ventricular outflow tract, and perivalvular leakage may also occur after prosthetic valve replacement and increase the risk of re-operation [19, 20].

The David procedure provides good short-term and long-term effects [21], although it is a complex and technically challenging procedure. This procedure is considered difficult for our hospital’s situation as most patients with acute DeBakey I AD were transported to our hospital from throughout the region and often arrived during the night. Thus, emergency surgery was often performed overnight by the team of surgeons that were on duty, and David’s procedure was considered impractical because of the surgical complexity and poor general condition of the patients, who are frequently have co-existing diseases. To address these issues, we developed a patch neointima technique that provides a short-term and mid-term curative effect [8, 10]. Moreover, this technique was relatively simple, provides good hemostasis and surgical outcomes, and has a short learning curve, which has allowed many surgeons to adopt and widely perform it at our hospital.

The present study compared the mid-term outcomes of the VSARR and the Bentall procedure as emergency treatment for middle-aged Chinese patients with acute DeBakey I AD. Acute DeBakey I AD is associated with systemic coagulation abnormalities, which leads to a large-scale consumption of platelets and prothrombin complexes, as well as AD lesions, severe vascular wall edema, and brittle vascular tissues. And some patients have various risks associated with their frequent co-existence diseases, and poor physical condition. Moreover, These factors increase intraoperative bleeding and the technical challenges of the procedure [22]. We did not encounter any uncontrolled bleeding during the operations, and esophageal ultrasonography revealed good repair at the aortic root. We attribute the good short-term outcomes for the patch neointima technique to its technical simplicity and short learning curve.

We did not detect any significant differences between the two groups in terms of operative time, CPB time, AC time, SBP time, intraoperative blood loss, intraoperative RBC infusionLow cardiac output syndrome, 24 h pericardial drainage volume, or re-thoracotomy for hemostasis. The advantages of the patch neointima technique are a simple procedure during which the patch is sutured to the aortic root, with proximal aortic anastomosis above the aortic valve connection. This technique provides a better surgical field and may help facilitate intraoperative hemostasis. Thus, the patch neointima technique may be useful for a broad range of root replacement surgeries in terms of shortening the operative time, reducing intraoperative bleeding, reducing the volume of blood products used, and reducing surgical complications, especially in younger patients.

The present study was also unable to detect any significant differences in postoperative neurological complications, acute renal failure, acute liver failure, gastrointestinal bleeding, sepsis, or spinal cord injury. It is possible that use of a triple-branched stent for total arch replacement helped reduce the postoperative complications in our patients [23]. This could be because the procedure is short, simple, requires fewer anastomoses, and provides a broad repair range, which could also contribute to reductions in the operative time, AC time, and SBP time. These factors greatly reduce the incidences of surgical mortality and postoperative complications.

In cases of acute DeBakey I AD, the major controversy is regarding whether the aortic valve should be preserved or replaced, as there are limited data regarding long-term survival and valvular complications in these patients. One report indicated that patients with acute DeBakey I AD who underwent valve-sparing survival had limited long-term survival, moderate re-operation risk, and a low risk of valve-related complications [3]. A meta-analysis by Benedetto et al. also raised concerns regarding long-term durability of valve-sparing repair [24]. However, none of our patients in group A required re-operation for AR during the follow-up, which suggests that the aortic valve-sparing patch technique helped maintain the valve’s function with only a mild expansion. (Table 3) Some reports have indicated that the Bentall procedure for acute DeBakey I AD provides reliable long-term survival, albeit with a considerable incidence of valvular complications [25, 26]. We also found that group B had a significantly increased postoperative aortic valve re-operation rate (1/111 patients [0.9%] vs. 8/90 patients [8.9%], *P* = 0.020) because of the anticoagulation-related thromboembolism/bleeding events. A patient who with a mechanical valve should oral warfarin anticoagulant therapy for lifelong. Therefore, the descending aorta fasle lumen was difficult to thromboticand and associated with a higher rate of endoleakage at the distal end of the triple-branched stent (0/111 patients [0.0%] vs. 6/90 patients [6.7%], *P* = 0.022). These outcomes support the existing concerns regarding long-term outcomes of the Bentall procedure, which suggest that aortic valve-sparing repair may be useful for managing the aortic root in acute DeBakey I AD cases. In this setting, the mechanical valve need for oral warfarin therapy, which is associated with various adverse effects.

### Limitations

This study involved a single-center retrospective analysis of a small sample of patients over a relatively short follow-up interval. These factors are associated with risks of bias.

## Conclusion

Among middle-aged Chinese patients with acute DeBakey I AD, VSARR were associated with a lower rate of endoleakage at the distal end of the triple-branched stent, thromboembolism/bleeding events and aortic valve re-operation, relative to the Bentall procedure.

## Data Availability

The data of this study will not be shared publically because they will be applied for further researches of this series. But authors do agree that the data can be shared individually if requested.

## Abbreviations

AD: Aortic dissection
VSARR: Modified Valve-sparing aortic root replacement technique
CTA: Computed tomography angiography
AR: Aortic regurgitation
EF: Left ventricular ejection fraction
CPB: Cardiopulmonary bypass
SBP: Selective brain perfusion
RBC: Red blood cell
